# Constitutive Tor2 Activity Promotes Retention of the Amino Acid Transporter Agp3 at Trans-Golgi/Endosomes in Fission Yeast

**DOI:** 10.1371/journal.pone.0139045

**Published:** 2015-10-08

**Authors:** Qingbin Liu, Yan Ma, Xin Zhou, Tomoyuki Furuyashiki

**Affiliations:** 1 Division of Pharmacology, Kobe University Graduate School of Medicine, Kobe, Japan; 2 Department of Oncology, the First Affiliated Hospital of Liaoning Medical University, Jinzhou, China; Kinki University School of Pharmaceutical Sciences, JAPAN

## Abstract

Amino acid transporters are located at specific subcellular compartments, and their localizations are regulated by the extracellular availability of amino acids. In yeast, target of rapamycin (TOR) activation induces the internalization of amino acid transporters located at the plasma membrane. However, whether and how TOR signaling regulates other amino acid transporters located at intracellular compartments remains unknown. Here, we demonstrate that in the fission yeast, the TOR inhibitor Torin–1 induces the transfer of several yellow fluorescent protein (YFP)-fused intracellular amino acid transporters, including Agp3, Isp5, Aat1, and Put4, from trans-Golgi/endosomes into the vacuoles. By contrast, the localizations of YFP-fused Can1, Fnx1, and Fnx2 transporter proteins were unaffected upon Torin–1 treatment. There are two TOR isoforms in fission yeast, Tor1 and Tor2. Whereas *tor1* deletion did not affect the Torin-1-induced transfer of Agp3-YFP, Tor2 inhibition using a temperature-sensitive mutant induced the transfer of Agp3-YFP to the vacuolar lumen, similar to the effects of Torin–1 treatment. Tor2 inhibition also induced the transfer of the YFP-fused Isp5, Aat1, and Put4 transporter proteins to the vacuoles, although only partial transfer of the latter two transporters was observed. Under nitrogen depletion accompanied by reduced Tor2 activity, Agp3-YFP was transferred from the trans-Golgi/endosomes to the plasma membrane and then to the vacuoles, where it was degraded by the vacuolar proteases Isp6 and Psp3. Mutants with constitutively active Tor2 showed delayed transfer of Agp3-YFP to the plasma membrane upon nitrogen depletion. Cells lacking Tsc2, a negative regulator of Tor2, also showed a delay in this process in a Tor2-dependent manner. Taken together, these findings suggest that constitutive Tor2 activity is critical for the retention of amino acid transporters at trans-Golgi/endosomes. Moreover, nitrogen depletion suppresses Tor2 activity through Tsc2, thereby promoting the surface expression of these transporters.

## Introduction

Eukaryotic cells utilize amino acid transporters with 12 transmembrane segments for the uptake of amino acids from the extracellular milieu. Dozens of amino acid transporters have been identified to date, each with specific substrates as well as unique subcellular localizations [1]. Several amino acid transporters are constitutively present at the cell surface where they mediate the basal uptake of amino acids. In contrast, other amino acid transporters are confined to intracellular compartments under nutrient-rich conditions, and are delivered to the cell surface upon nutrient deprivation for efficient amino acid uptake [2, 3]. Lower eukaryotes such as the budding yeast *Saccharomyces cerevisiae* and the fission yeast *Schizosaccharomyces pombe* have been frequently used as model organisms to investigate the molecular mechanism regulating the properties of amino acid transporters. These studies have all pointed to an important role of target of rapamycin (TOR) signaling in amino transporter regulation and translocation.

TOR is a serine/threonine kinase that is highly conserved across species; it regulates cellular growth and proliferation in response to nutritional status, and its abnormality has been implicated in various human diseases [4, 5]. TOR functions as a core kinase of either of two types of protein complexes, namely TORC1 and TORC2, which are differently regulated and play distinct roles. Mammalian cells have only a single TOR kinase for both mTORC1 and mTORC2, whereas yeast cells have two TOR isoforms for TORC1 and TORC2, respectively. Genetic studies using yeast have revealed the roles of TOR signaling in adaptive responses upon nitrogen deprivation, such as in G1 cell cycle arrest and sexual development [6, 7]. In this process, nitrogen deprivation suppresses TOR signaling, which in turn disinhibits the GATA-mediated transcription of various genes, including several amino acid transporters [8, 9].

Further studies in yeast have suggested a novel role of TORC1 in regulating the subcellular localization of amino acid transporters. In budding yeast, cyclohemixide, a drug that stimulates TORC1, induces internalization of the amino acid transporter Can1p from the plasma membrane in a rapamycin-sensitive manner [2]. Likewise, in fission yeast, deletion of *tsc2*, a negative regulator of TORC1, induces the internalization of the amino acid transporter Cat1 from the plasma membrane [10, 11]. Therefore, it is established that TORC1 overstimulation can induce the internalization of surface amino acid transporters, although the physiological relevance of these findings is uncertain. By contrast, the role of TORC1 in regulating the localizations of intracellular amino acid transporters remains elusive. It is known that nitrogen depletion induces the transfer of intracellular amino acid transporters, if not all transporters, to the cell surface. Since nitrogen depletion suppresses TORC1 activity, it is plausible that TORC1 suppression is the underlying mechanism contributing to the observed surface transfer of amino acid transporters upon nitrogen depletion. Although deletion of *tsc2* was reported to reduce the surface expression of the amino acid transporter Aat1 under nitrogen depletion in fission yeast [3], the authors suggested that the action of Tsc2 is independent from that of Tor2, a core kinase of TORC1. In addition, whether and how TORC1 regulates the localizations of other intracellular amino acid transporters has not been examined.

In this study, using pharmacological and genetic approaches, we evaluated the role of Tor2 in the localizations of several intracellular amino acid transporters. We focused specifically on Agp3 as a representative of these Tor2-sensitive transporters, and our findings suggest that constitutive Tor2 activity promotes the retention of Agp3 at the trans-Golgi/endosomes, regardless of the availability of nitrogen sources.

## Materials and Methods

### Strains and Media

The *S*. *pombe* strains used in this study are listed in Table 1. Edinburgh minimal medium (EMM) was used as the nitrogen-rich culture medium, as described previously [12]. EMM without NH_4_Cl was used as the nitrogen-depleted medium.

**Table 1:** *Schizosaccharomyces pombe* Strains used in this Study.

Strain	Genotype	Reference
HM123	*h* ^*-*^ *leu1-32*	Our stock
KP207	*h* ^*+*^ *leu1 his2*	Our stock
KP456	*h* ^*-*^ *leu1-32 ura4-D18*	Our stock
KP928	*h* ^*+*^ *his2 leu1-32 ura4-D18*	Our stock
KP1245	*h* ^*+*^ *leu1-32 ura4-294*	Our stock
KP2649	*h* ^*-*^ *leu1 ura4-294 pREP42-*Krp1-RFP:*ura4* ^*+*^	Our stock
KP5080	*h* ^*-*^	[13]
KP6153	*h* ^*-*^ *leu1-32 pREP1-*Isp5-YFP:*leu1* ^*+*^	This study
KP6154	*h* ^*-*^ *leu1-32 pREP1-*Agp3-YFP:*leu1* ^*+*^	This study
KP6155	*h* ^*-*^ *leu1-32 pREP1-*Put4-YFP:*leu1* ^*+*^	This study
KP6156	*h* ^-^ *leu1-32 pREP1-*Aat1-YFP:*leu1* ^*+*^	This study
KP6157	*h* ^*-*^ *leu1-32 pREP1-*Fnx1-YFP:*leu1* ^*+*^	This study
KP6158	*h* ^*-*^ *leu1-32 pREP1-*Can1-YFP:*leu1* ^*+*^	This study
KP6159	*h* ^*-*^ *leu1-32 pREP1-*Fnx2-YFP:*leu1* ^*+*^	This study
KP5131	*h* ^*-*^ *leu1-32 tsc2*::*KanMX*_*4*_	[13]
KP6434	*h* ^*-*^ *leu1-32 tsc2*::*KanMX*_*4*_ *pREP1-*Agp3-YFP:*leu1* ^*+*^	This study
KP3222	*h* ^*-*^ *leu1-32 ura4-D18 tor1*::*ura4* ^*+*^	Our stock
KP6239	*h* ^*-*^ *leu1-32 ura4-D18 tor1*::*ura4* ^*+*^ *pREP1-*Agp3-YFP:*leu1* ^*+*^	This study
KP5482	*h* ^*-*^ *leu1-32 tor2-287*	[14]
KP6244	*h* ^*-*^ *leu1-32 tor2-287 pREP1-*Agp3-YFP:*leu1* ^*+*^	This study
KP6448	*h* ^*-*^ *leu1-32 tor2-287 tsc2*::*KanMX*_*4*_ *pREP1-*Agp3-YFP:*leu1* ^*+*^	This study
KP6624	*h* ^*-*^ *leu1-32 ura4-294 pREP1-*Agp3-YFP:*leu1* ^*+*^ *pREP42-*Krp1-RFP:*ura4* ^*+*^	This study
KP6621	*h* ^+^ *his2 leu1-32 ura4-D18 pREP1-*Agp3-YFP:*leu1* ^*+*^	This study
KP6642	*h* ^+^ *leu1-32 ura4-294 pREP42-*Agp3-RFP:*ura4* ^*+*^	This study
KP5873	*h* ^*90*^ *tor2* ^L1310P^::*KanMX*_*4*_ (JUP1350)	[15]
KP6637	*h* ^*-*^ *leu1-32 tor2* ^L1310P^::*KanMX*_*4*_ *pREP1-*Agp3*-YFP*:*leu1* ^*+*^	This study
KP5874	*h* ^*90*^ *tor2* ^E2221K^::*KanMX*_*4*_ (JUP1352)	[15]
KP6638	*h* ^*-*^ *leu1-32 tor2* ^E2221K^::*KanMX*_*4*_ *pREP1*-Agp3-YFP:*leu1* ^*+*^	This study
KP6644	*h* ^*-*^ *leu1-32 ura4-294 pREP42-*Agp3-RFP:*ura4* ^*+*^ *pREP1-*Fnx1-YFP:*leu1* ^*+*^	This study
KP6651	*h* ^*-*^ *leu1-32 tor2-287 pREP1-*Isp5-YFP:*leu1* ^*+*^	This study
KP6652	*h* ^*-*^ *leu1-32 tor2-287 pREP1-*Put4-YFP:*leu1* ^*+*^	This study
KP6653	*h* ^*-*^ *leu1-32 tor2-287 pREP1-*Aat1-YFP:*leu1* ^*+*^	This study
KP5857	*h* ^*+*^ *leu1-32 tor2-287*	Our stock
KP6659	*h* ^*+*^ *his2 leu1-32 ura4-294 pREP42-*Krp1-RFP:*ura4* ^*+*^	This study
KP6662	*h* ^*-*^ *leu1-32 ura4-294 pREP1-*Isp5-YFP:*leu1* ^*+*^ *pREP42-*Krp1-RFP:*ura4* ^*+*^	This study
KP6664	*h* ^*-*^ *leu1-32 ura4-294 pREP1-*Put4-YFP:*leu1* ^*+*^ *pREP42-*Krp1-RFP:*ura4* ^*+*^	This study
KP6665	*h* ^*-*^ *leu1-32 ura4-294 pREP1-*Aat1-YFP:*leu1* ^*+*^ *pREP42-*Krp1-RFP:*ura4* ^*+*^	This study
KP2888	*h- leu1-32 ura4-D18 isp6*::*ura4* ^*+*^ *psp3*::*ura4* ^*+*^	[16]
KP6635	*h- leu1-32 ura4-D18 isp6*::*ura4* ^*+*^ *psp3*::*ura4* ^*+*^ *pREP1-*Agp3-YFP:*leu1* ^*+*^	This study

### Construction of *S*. *pombe* Strains Expressing Yellow Fluorescent Protein (YFP)- or Red Fluorescent Protein (RFP)-fused Amino Acid Transporters

To create yeast strains expressing YFP-fused Agp3, the pDUAL-YFH1c plamid [17] containing Agp3-YFP-FLAG-His6 (Gene ID 25/H04) was purchased from the DNA Bank of the RIKEN BioResource Center. The plasmid was digested with *Not*I and the resultant fragment was integrated into the *leu1* ^*+*^ locus of the strain HM123 (*h* ^-^ *leu1-32*). Leu^+^ transformants were selected on EMM plates with 4 μM thiamine. Strains expressing YFP-fused Isp5, Put4, Aat1, Fnx1, Can1, and Fnx2 were similarly created with corresponding expression plasmids (Gene IDs 35/H09, 34/G09, 45/F12, 33/C04, 48/A04, 35/G09, respectively) obtained from the DNA Bank of the RIKEN BioResource Center.

To create strains expressing RFP-fused Agp3, the *agp3* ^+^ gene was amplified by polymerase chain reaction from the genomic DNA of wild-type cells as a template. The sense primer was (#5012) 5′-GAA GAT CTA TGG AGG CAG CGC CTT CTG–3′, and the antisense primer was (#5013) 5′-ATA AGA ATG CGG CCG CAT ACT TCT TTC CAA ACT C–3′. The amplified product containing the *agp3* ^+^ gene was digested with *Bgl*II and *Not*I, and the resultant fragment containing the *agp3* ^+^ gene was inserted to the C terminus of the RFP vector under the *nmt42* promoter carrying the *ura4* ^*+*^ marker. The plasmid was digested with *Stu*I, and the resultant fragment was integrated into the *ura4* ^*+*^ locus of the strain KP1245 (*h* ^*+*^ *leu1-32 ura4-294*). Ura^+^ transformants were selected on EMM plates with 4 μM thiamine.

### FM4-64 Staining

To visualize the vacuolar membranes, FM4-64 staining was performed as described previously [18]. In brief, the cells were grown to the log phase in EMM at 27°C, harvested, and resuspended in EMM. FM4-64 was added at 40 μM, and the cells were incubated at 27°C for 40 min. The cells were then harvested and washed twice in EMM to remove free FM4-64. The cells were resuspended in EMM and further incubated at 27°C for 1 h before fluorescent imaging.

### Fluorescent Imaging

Microscopic observations were performed as described previously [19]. To induce expression of the YFP/RFP-fused amino acid transporters, the yeast strains described above were grown without thiamine for 18–20 h. In most cases, the live cells were mounted on glass slides and subjected to microscopic analysis using an Axioskop 2 Plus microscope (Carl Zeiss, Inc., Germany) equipped with an alpha Plan-Fluor 100x/N.A.1.45 oil objective (Carl Zeiss, Inc.). To precisely assess the co-localization of YFP-fused amino acid transporters and Krp1-RFP, the cells were fixed with 2% paraformaldehyde and observed in the same manner described above. Fluorescent images were taken using a SPOT 2 digital camera in combination with the Spot32 software version 2.1.2 (Diagnostic Instruments, Sterling Heights, MI, USA). Fluorescent images were processed using Adobe Photoshop CS6 only for illustrative purposes.

### Protein Extraction and Western Blotting

Protein extraction and western blotting were performed as described previously [20] with minor modifications. To induce the expression of Agp3-YFP, the cells integrated with its expression cassette were grown to the log phase in EMM. The cells were lysed in 500 μL of NaOH lysis buffer (1.85 M NaOH, 7.5% β-mercaptoethanol) and incubated for 10 min on ice. Proteins were precipitated by adding 500 μL of 50% trichloroacetic acid and incubated for 10 min on ice. After centrifugation, the pellet was washed with 1 M Tris base twice, and solubilized in sodium dodecyl sulfate (SDS) sample buffer without bromophenol blue. Protein concentration was determined using Protein Assay BCA Kit (Wako) according to the manufacturer’s protocol. The absorbance at 562 nm was recorded on an Epoch 2 microplate spectrophotometer (Biotek). Samples containing 20 μg of proteins were subjected to SDS-polyacrylamide gel electrophoresis with Extra PAGE One Precast Gel (10%) (Nacalai) and transferred to a 0.45-μm polyvinylidene fluoride blotting membrane (Amersham Hybond, GE Healthcare). Agp3 was detected using primary green fluorescent protein antibody and secondary horseradish peroxidase-conjugated anti-rabbit IgG antibody (Cell Signaling). Immunoreactivity was visualized with Clarity Western ECL Substrate (Bio-Rad). After draining, the membranes were covered in clear plastic wrapping and exposed to FUJI medical X-ray film (RX-U, FUJI Film) for 0.5–30 min, depending on the strength of the signal.

## Results

### Tor2 Inhibition Induces the Transfer of Several Amino Acid Transporters from Trans-Golgi/Endosomes to the Vacuolar Lumen

In this study, we examined the role of TOR signaling in the localizations of several representative amino acid transporters whose functions have been relatively well characterized (Agp3, Isp5, Aat1, Put4, Can1, Fnx1, and Fnx2). Torin–1 is a potent and selective mTOR inhibitor in mammalian cells [21], and it inhibits the phosphorylation of Rps6 and Gad8, which are specific substrates for Tor2 and Tor1, respectively, in fission yeast [13, 22]. Using the cells expressing the YFP-fused amino acid transporters under the pREP1-inducible promoter integrated in the chromosome, we examined the effect of Torin–1 treatment at 400 nM on the localization of these amino acid transporters.

**Fig 1:**
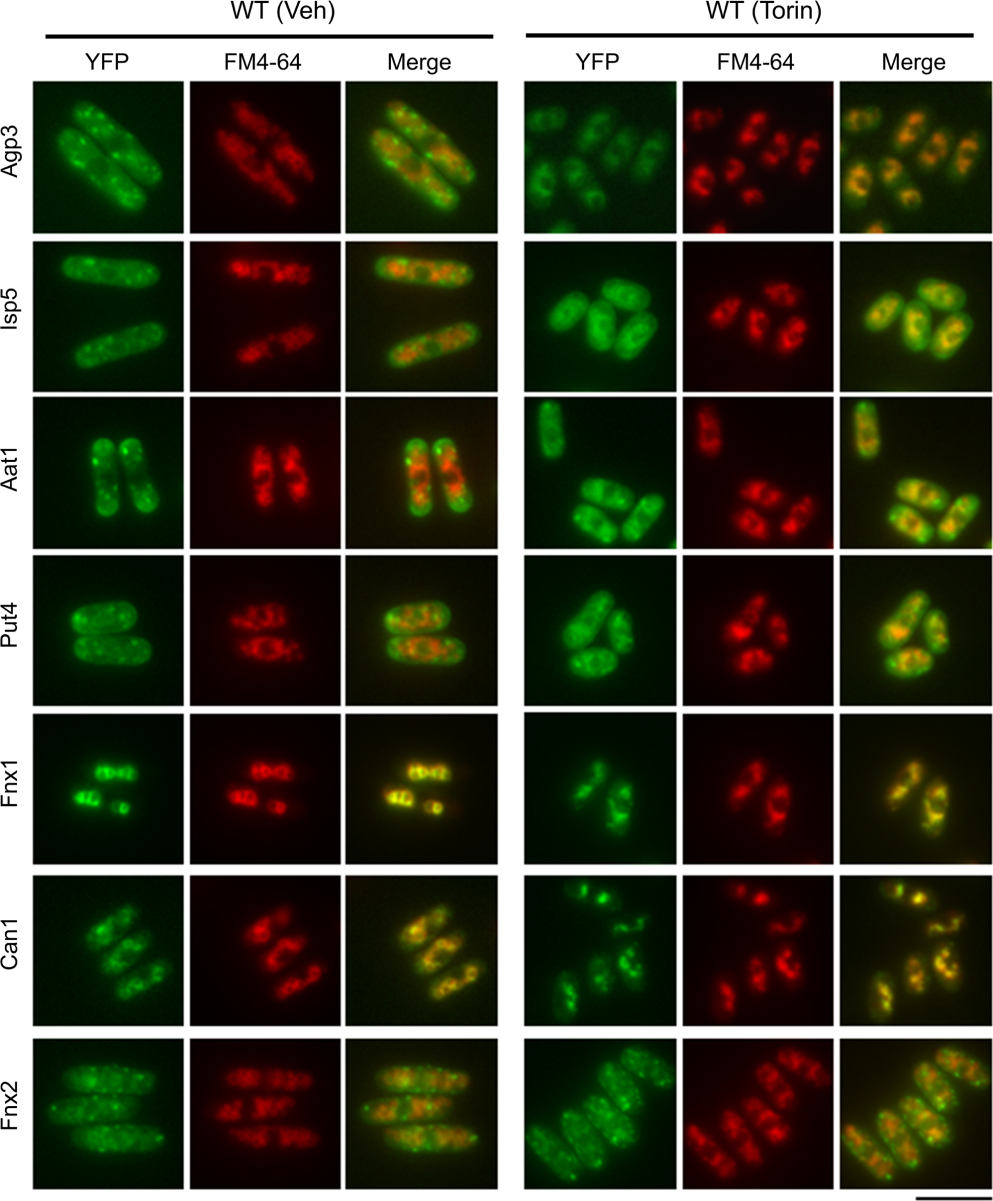
Systematic analysis of the localizations of several representative amino acid transporters with or without TOR inhibition in fission yeast. Representative fluorescent images of the wild-type (WT) cells expressing YFP-fused Agp3 (KP6154), Isp5 (KP6153), Aat1 (KP6156), Put4 (KP6155), Fnx1 (KP6157), Can1 (KP6158), or Fnx2 (KP6159) protein with vehicle (Veh) or Torin–1 (Torin) treatment for 2 h. The cells were grown to the log phase in EMM, and were subjected to vehicle or Torin–1 treatment. To visualize the vacuolar membranes, FM4-64 was loaded for 1 h before the Torin–1 treatment. YFP and FM4-64 signals are shown in green and red, respectively, and the merged signals are shown in yellow. Scale bar, 10 μm.

In the untreated condition, the YFP-fused Agp3, Isp5, Put4, and Fnx2 proteins were enriched, as indicated by cytoplasmic dot-like structures (Fig 1, Veh). Aat1-YFP was enriched at these cytoplasmic dot-like structures as well as at the cell periphery. Can1-YFP and Fnx1-YFP accumulated at vesicular structures larger than the cytoplasmic dot-like structures. To better visualize the intracellular structures, we loaded these cells with FM4-64 dye, which accumulates at the vacuolar membranes after 1 h of dye uptake. Can1-YFP and Fnx1-YFP, but not the other YFP-fused amino acid transporters, colocalized with circular FM4-64 signals (Fig 1, Veh), suggesting that these transporters are localized on the vacuolar membranes. Although Fnx2-YFP was not colocalized with FM4-64 in this experimental condition, it has previously been reported to be localized on the vacuolar membrane [23]. This difference may be due to the fact that nutrient-rich YES medium was used in the previous study, whereas EMM was used in this study. Indeed, we observed colocalization of Fnx2-YFP with circular FM4-64 signals, and thus on the vacuolar membrane, in YES medium (S1 Fig), as opposed to localization on the vesicular structures in EMM (Fig 1, veh). This observation suggests that the culture condition, perhaps reflecting the nutrient availability, affects the localization of Fnx2.

After Torin–1 treatment for 2 h, the YFP-fused Agp3, Isp5, Aat1, and Put4 proteins were localized in the FM4-64-positive structures, indicating their transfer into vacuoles, although the transfer of Aat1-YFP and Put4-YFP was only partial (Fig 1, Torin). In EMM, Torin–1 treatment results in the vacuole being too small to accurately discriminate between the membrane and lumen. Since the vacuole appears to be larger in YES medium than in EMM, we also examined the localization of Agp3 in YES medium with or without Torin–1 treatment. In this culture condition, Agp3-YFP was clearly observed in only the vacuolar lumen after Torin–1 treatment (S2 Fig). This finding suggests that Torin–1 treatment induces the transfer of several amino acid transporters, including Agp3, from the cytoplasmic dot-like structures to the vacuolar lumen. By contrast, Torin–1 treatment did not affect the localizations of Can1, Fnx1, or Fnx2 (Fig 1, Torin). Thus, under Torin–1 treatment, Can1-YFP and Fnx1-YFP still colocalized with the FM4-64-stained vacuolar membrane, although the size of the vacuoles was reduced. These results suggest that different amino acid transporters are localized at distinct subcellular compartments, and that TOR inhibition alters the localizations of several amino acid transporters, namely Agp3, Isp5, Aat1, and Put4.

**Fig 2:**
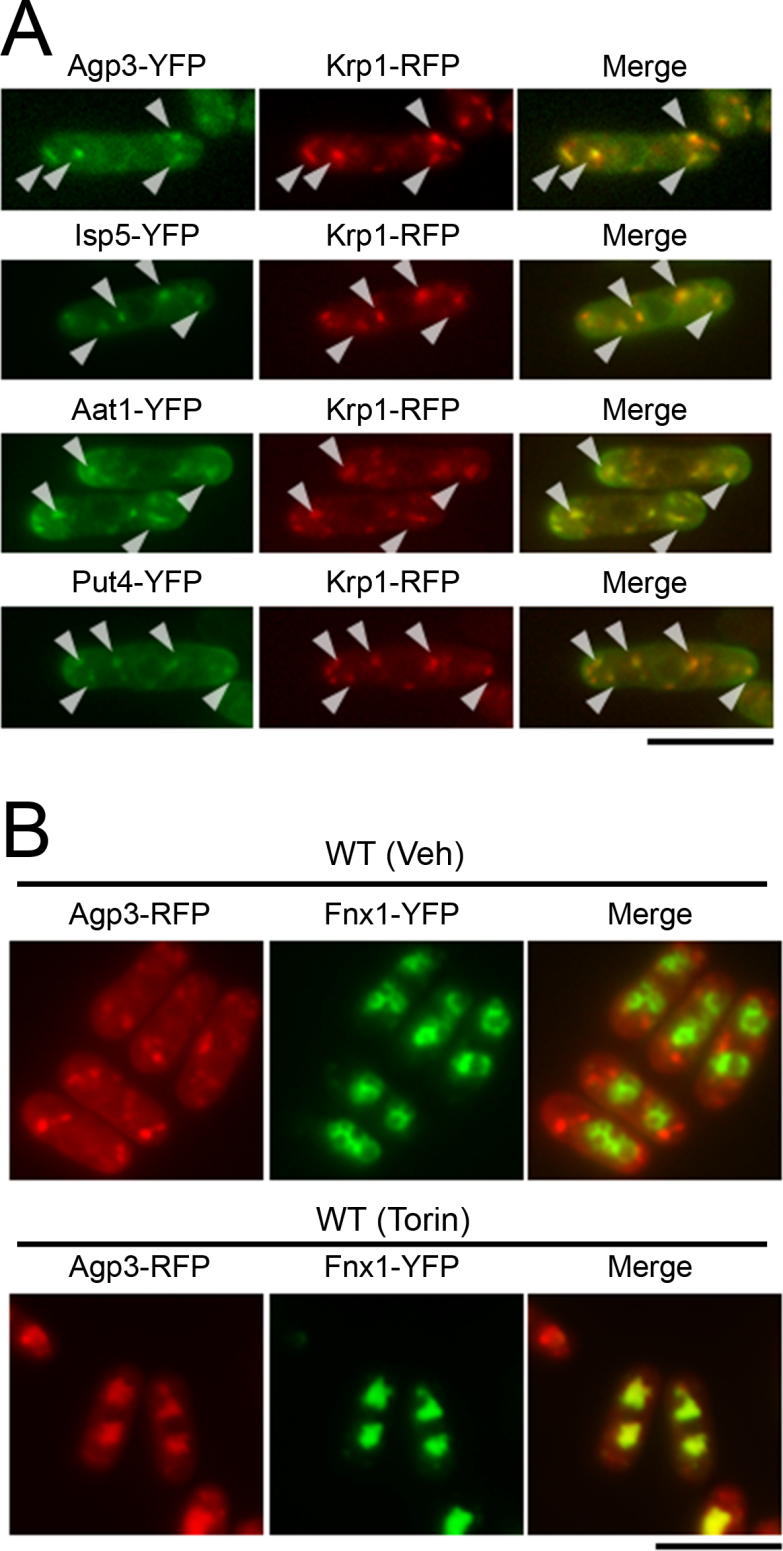
TOR inhibition induces the transfer of Agp3, Isp5, Aat1, and Put4 from trans-Golgi/endosomes into the vacuoles. (A) The co-localization of Agp3, Isp5, Aat1, and Put4 with Krp1, a marker for trans-Golgi/endosomes, in normal culture condition. Representative fluorescent images of the wild-type cells co-expressing Krp1-RFP and YFP-fused Agp3 (KP6624), Isp5 (KP6662), Aat1 (KP6665), or Put4 (KP6664) protein are shown. YFP and RFP signals are shown in green and red, respectively, and the merged signals are yellow. Arrowheads indicate the co-localization of Agp3-positive dots with the Krp1-positive structures. Scale bar, 10 μm. (B) The co-localization of Agp3 with Fnx1, a vacuole-enriched amino acid transporter, after Torin–1 treatment. Representative fluorescent images of the wild-type cells co-expressing Agp3-RFP and Fnx1-YFP (KP6644) with vehicle (Veh) or Torin–1 (Torin) treatment for 2 h. RFP and YFP signals are shown in red and green, respectively, and the merged signals are yellow. Scale bar, 10 μm.

To examine whether the cytoplasmic dot-like structures of the Torin-1-sensitive transporters represent trans-Golgi/endosomes, we investigated the co-localization of these transporters with Krp1, an endopeptidase that is cycled between the trans-Golgi and endosomal compartments [24]. In the normal culture condition, YFP-fused Agp3, Isp5, Aat1, and Put4 proteins were co-localized with Krp1-RFP (Fig 2A, arrowheads), suggesting the localization of these transporters at the trans-Golgi/endosomes. We also generated cells expressing both Agp3-RFP and Fnx1-YFP, the latter of which was colocalized on the vacuolar membrane as described above (Fig 1). In the untreated condition, the Agp3-positive dots were not contained in the Fnx1-positive vacuoles (Fig 2B, Veh), whereas Torin–1 treatment induced the transfer of the Agp3 signals to Fnx1-positive vacuoles, perhaps to the vacuolar lumen (Fig 2B, Torin). These findings suggest that Torin–1 treatment induces the transfer of Agp3 as well as Isp5, Aat1, and Put4 from the trans-Golgi/endosomes into the vacuoles.

**Fig 3:**
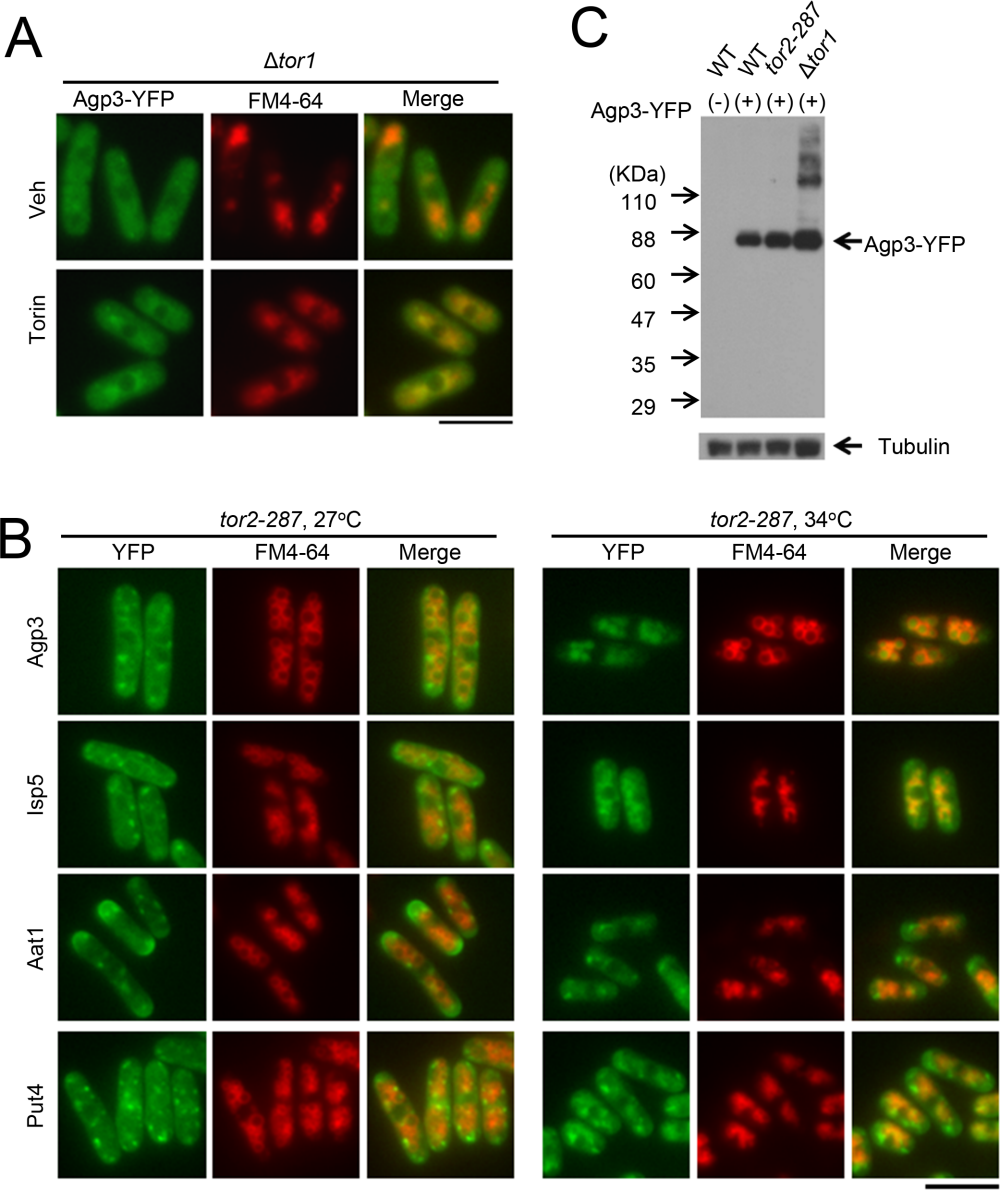
Tor2 inhibition induces the transfer of Agp3, Isp5, Aat1, and Put4 from trans-Golgi/endosomes into the vacuoles. (A) The effect of *tor1* deletion on the localization of amino acid transporters. Representative fluorescent images of Δ*tor1* cells expressing Agp3-YFP (KP6239) with vehicle (Veh) or Torin–1 (Torin) treatment for 2 h are shown. To visualize the vacuolar membranes, FM4-64 was loaded for 1 h before the Torin–1 treatment. YFP and FM4-64 signals are shown in green and red, respectively, and the merged signals are yellow. Scale bar, 10 μm. (B) The effect of *tor2-287* mutation on the localization of amino acid transporters. Representative fluorescent images of *tor2-287* cells expressing Agp3-YFP (KP6244), Isp5 (KP6651), Aat1 (KP6653), or Put4 (KP6652) with vehicle (Veh) or Torin–1 (Torin) treatment for 2 h. Scale bar, 10 μm. (C) Expression of intact Agp3-YFP protein in the strains used in this study. The wild-type cells (KP6154, WT(+)), *tor2-287* cells (KP6244), and Δ*tor1* cells (KP6239) expressing Agp3-YFP, as well as the wild-type cells without Agp3-YFP expression (KP5080, WT(-)), were collected, and the proteins were extracted for western blotting. Agp3-YFP was detected using the GFP antibody. Tubulin expression was used as an internal control.

Given that there are two TOR isoforms, Tor1 and Tor2, we examined the localization of Agp3-YFP in Δ*tor1* and temperature-sensitive *tor2* mutant cells (*tor2-287*) [14]. In Δ*tor1* cells, Agp3-YFP showed a diffuse cytoplasmic distribution, in contrast to the cytoplasmic dot-like structures observed in wild-type cells. However, in Δ*tor1* cells, Torin–1 treatment still induced the transfer of Agp3-YFP into FM4-64-stained vacuoles (Fig 3A), similar to the effects observed in wild-type cells. In *tor2-287* cells, Agp3-YFP showed cytoplasmic dot-like signals at the permissive temperature (27°C). However, when the cells were cultured at the restrictive temperature (34°C) for 2 h to inhibit Tor2 activity, Agp3-YFP was transferred into the lumen of FM4-64-stained vacuoles (Fig 3B). This transfer of Agp3-YFP from cytoplasmic dot-like structures to the vacuolar lumen was not observed in wild-type cells after culture at 34°C for 2 h (data not shown), thereby confirming the effect of Tor2 inhibition on Agp3 localization. The immunoblotting results confirmed the expression of intact Agp3 proteins in wild-type cells, Δ*tor1* cells, and *tor2-287* cells, although higher-molecular-weight species of Agp3-YFP were also present in Δ*tor1* cells (Fig 3C). Collectively, these findings suggest that Torin–1 treatment induces the transfer of Agp3 from trans-Golgi/endosomes to the vacuolar lumen through Tor2 inhibition. It should be noted that Torin–1 treatment and *tor1* deletion both decrease the size of the vacuoles, whereas Tor2 inhibition via *tor2-287* mutation increases the vacuole size (Fig 1, Torin; Fig 3). These findings suggest that Tor1 and Tor2 play opposite roles in regulating the size of the vacuoles in fission yeast.

**Fig 4:**
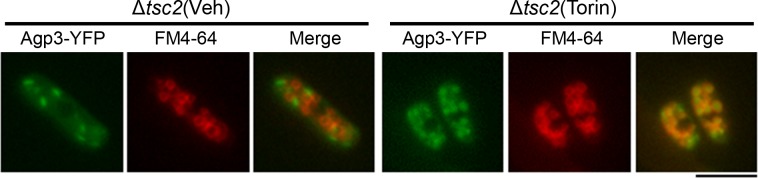
Constitutive Tor2 activity is sufficient for Agp3 localization at trans-Golgi/endosomes. Representative images of Δ*tsc2* cells expressing Agp3-YFP with vehicle (Veh) or Torin–1 (Torin) treatment are shown. The cells were grown to the log phase in EMM, and subjected to drug treatment. In this experiment, Torin–1 treatment for 4 h rather than 2 h was required for it to exert its effect, which was perhaps due to the elevated Tor2 activity induced by *tsc2* deletion. YFP and FM4-64 signals are shown in green and red, respectively, and the merged signals are yellow. Scale bar, 10 μm.

Given the apparent role of Tor2 in regulating Agp3 localization, we next examined whether elevated Tor2 activity induced by *tsc2* deletion might affect Agp3-YFP localization. In Δ*tsc2* cells, Agp3-YFP showed normal cytoplasmic dot-like structures, and Torin–1 treatment induced the transfer of Agp3-YFP into the vacuolar lumen (Fig 4), as seen in wild-type cells (Fig 1). Therefore, constitutive Tor2 activity is sufficient for maintaining Agp3 localization at trans-Golgi/endosomes.

Next, we examined whether Tor2 is similarly involved in the localizations of other amino acid transporters enriched in the cytoplasmic dot-like structures, namely Isp5, Aat1, and Put4 (Fig 3B). These YFP-fused amino acid transporters showed similar distributions in *tor2-287* cells as observed in wild-type cells at the permissive temperature (27°C). Tor2 inhibition at the restrictive temperature (34°C) for 2 h induced the transfer of Isp5, Aat1, and Put4 from trans-Golgi endosomes into the vacuoles (Fig 3B). Consistent with the effect of Torin–1 treatment described above (Fig 1), the transfer of Aat1-YFP and Put4-YFP to the vacuoles was only partial with Tor2 inhibition for 2 h (Fig 3B) and 6 h (data not shown). Therefore, Tor2 regulates the localizations of several, if not all, amino acid transporters, and promotes the retention of these transporters at the trans-Golgi/endosomes.

### Decreased Tor2 Activity upon Nitrogen Depletion Promotes the Surface Localization of Agp3 through Tsc2

**Fig 5:**
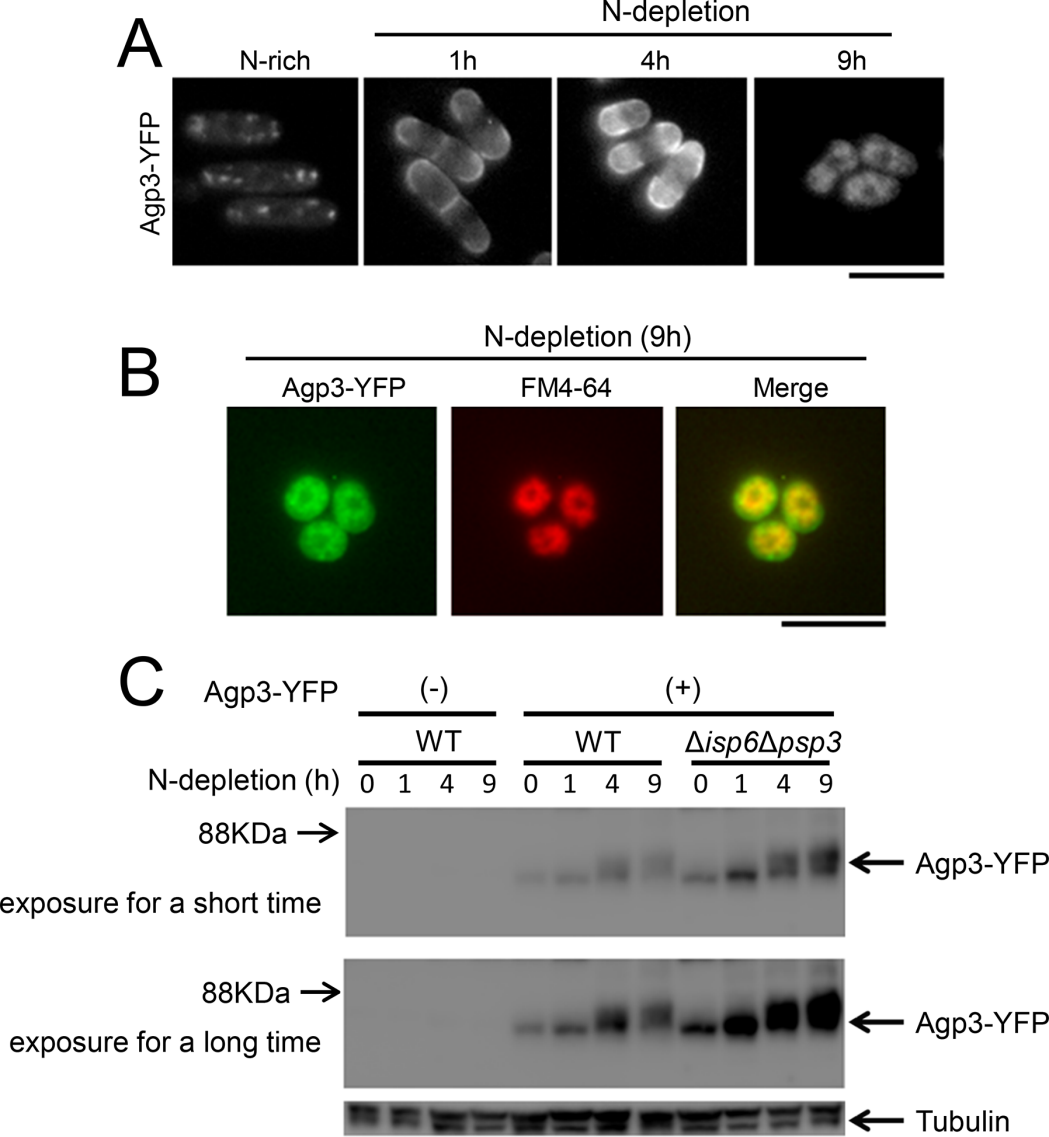
Nitrogen depletion induces the transfer of Agp3 from trans-Golgi/endosomes to the plasma membrane, and then into the vacuoles. (A) The effect of nitrogen depletion on Agp3 localization. Representative images of wild-type cells expressing Agp3-YFP (KP6154) in nitrogen-rich medium (N-rich) or after the shift to nitrogen-depleted medium for 1 h, 4 h, and 9 h are shown. Scale bar, 10 μm. (B) The localization of Agp3 in FM4-64-stained vacuoles under nitrogen depletion for 9 h. Wild-type cells expressing Agp3-YFP were cultured in nitrogen-depleted medium for 8 h, and FM6-64 was loaded for 1 h in recycled nitrogen-depleted medium to avoid nutrient re-supplementation. Scale bar, 10 μm. (C) The expression of Agp3-YFP and its degradation by vacuolar proteases under nitrogen depletion. The wild-type cells (KP6154, WT, (+)) and Δ*isp6*Δ*psp3* cells (KP6635, Δ*isp6*Δ*psp3*, (+) expressing Agp3-YFP were collected without (0 h) or with nitrogen depletion (1 h, 4 h, and 9 h), and the proteins were extracted for western blotting. Agp3-YFP was detected with green fluorescent protein antibody. The wild-type cells without Agp3-YFP expression (KP5080, WT, (-)) were similarly analyzed as negative controls. The same immunoblot is shown with shorter and longer exposures. Tubulin expression was used as an internal control.

In fission yeast, nitrogen depletion downregulates Tor2 activity [6, 7, 25]. This phenomenon prompted us to examine the localization of Agp3 upon nitrogen depletion. Nitrogen depletion induced the transfer of Agp3-YFP from the trans-Golgi/endosomes to the plasma membrane within 1 h (Fig 5A, N-depletion, 1 h and 4 h), which then accumulated in FM4-64-stained vacuoles at no later than 9 h (Fig 5A and 5B, N-depletion, 9 h). The immunoblotting results confirmed the expression of Agp3-YFP at its expected molecular weight in both nitrogen-rich and nitrogen-depleted medium (Fig 5C, Agp3-YFP (+), WT). The amount of Agp3-YFP appeared to be reduced after nitrogen depletion for 9 h, suggesting that its transfer to the vacuoles is associated with its degradation. Given the localization of Agp3-YFP in the vacuole, we hypothesized that vacuolar proteases might be involved in the process of Agp3-YFP degradation. There are at least two proteases that are known to function in the vacuole, Isp6 and Psp3 [16]. We therefore examined whether these vacuolar proteases are involved in regulating the protein level of Agp3-YFP. The amounts of Agp3-YFP proteins increased in Δ*isp6*Δ*psp3* cells in both nitrogen-rich and nitrogen-depleted conditions (Fig 5C). This increase was particularly obvious after nitrogen depletion for 9 h, in which Agp3 was mostly localized inside the vacuole. This result suggests that the transfer of Agp3 into the vacuole under nitrogen depletion conditions is coupled with its degradation mediated by Isp6 and Psp3.

**Fig 6:**
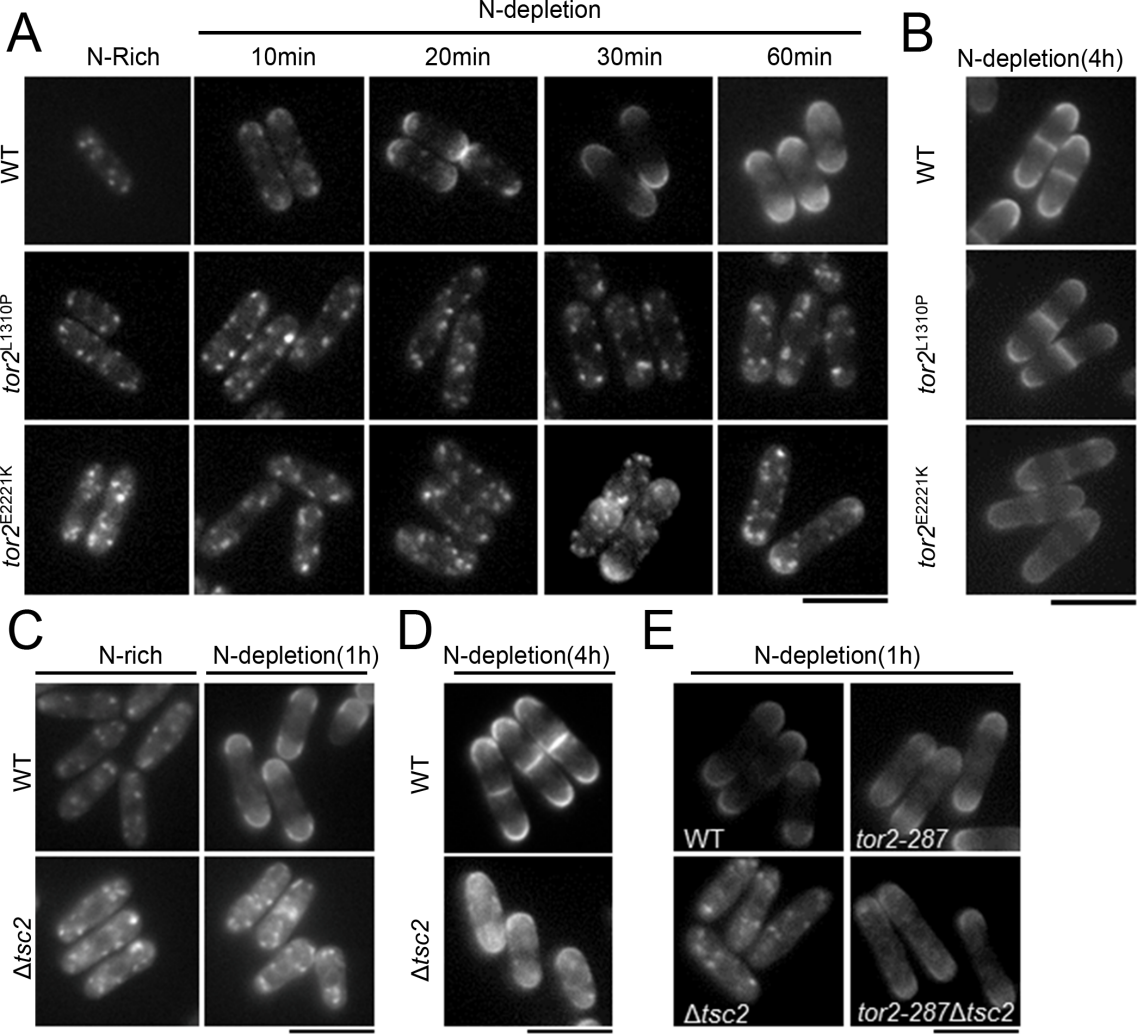
Increased Tor2 activity delays the transfer of Agp3 to the plasma membrane under nitrogen depletion. (A, B) The effect of constitutively active *tor2* mutants on the transfer of Agp3 to the plasma membrane under nitrogen depletion. Representative fluorescent images of wild-type cells (KP6154, WT), *tor2* ^L1310P^ cells (KP6637), and *tor2* ^E2221K^ cells (KP6638) expressing Agp3-YFP in nitrogen-rich medium (N-rich) or after a shift to nitrogen-depleted medium (N-depletion) for 10 min, 20 min, 30 min, and 60 min (A), and of the same cells of another batch under nitrogen depletion for 4 h (B). Scale bar, 10 μm. (C, D) The effect of *tsc2* deletion on the transfer of Agp3 to the plasma membrane under nitrogen depletion. Representative fluorescent images of the wild-type cells (KP6154, WT) and Δ*tsc2* cells (KP6434) expressing Agp3-YFP in nitrogen-rich medium (N-rich) or after the shift to nitrogen-depleted medium (N-depletion) for 1 h (C) and 4 h (D). Scale bar, 10 μm. (E) Rescue of the nitrogen depletion-induced transfer of Agp3 to the plasma membrane in Δ*tsc2* cells by the *tor2-287* mutation. Representative images of the wild-type cells (KP6154), Δ*tsc2* cells (KP6434), *tor2-287* cells (KP6244), and *tor2-287*Δ*tsc2* cells (KP6448) expressing Agp3-YFP under nitrogen depletion for 1 h are shown. Scale bar, 10 μm.

To examine whether decreased Tor2 activity is involved in the nitrogen depletion-induced change in Agp3 localization, we evaluated this process in two constitutively active Tor2 mutants, *tor2* ^L1310P^ and *tor2* ^E2221K^ [15]. These Tor2 mutants have been shown to confer Rhb1-independent growth [15] and elevated Tor2 activity using Rsp6 as a readout [26]. In wild-type cells, Agp3-YFP started to transfer to the plasma membrane as early as 10 min after the shift to the nitrogen-depletion condition, and the transfer was completed after 30 min and maintained for up to at least 4 h (Fig 6A and 6B, WT). In contrast, in the *tor2* ^L1310P^ and *tor2* ^E2221K^ mutants, the transfer of Agp3-YFP to the plasma membrane was impaired with nitrogen depletion for up to 1 h (Fig 6A, *tor2* ^L1310P^ and *tor2* ^E2221K^), whereas Agp3-YFP was completely transferred to the plasma membrane after 4 h in the wild-type and both mutants (Fig 6B). These findings suggest that constitutive Tor2 activity delays the surface expression of Agp3 upon nitrogen depletion.

Since Tsc2 is a negative regulator of Tor2, we examined its involvement in the nitrogen depletion-induced change in Agp3 localization. In Δ*tsc2* cells, Agp3-YFP failed to transfer from the trans-Golgi/endosomes to the plasma membrane within 1 h after the shift to nitrogen depletion medium, but did complete the transfer after 4 h (Fig 6C and 6D). Since *tsc2*-deficient cells showed a similar deficit to that observed in the above Tor2 active mutants, we investigated whether elevated Tor2 activity underlies the defective transfer of Agp3-YFP observed under nitrogen depletion in Δ*tsc2* cells. In *tor2-287*Δ*tsc2* cells, the transfer of Agp3-YFP from trans-Golgi/endosomes to the plasma membrane was restored within 1 h after the transfer to nitrogen-depleted medium (Fig 6E). This result suggested that elevated Tor2 activity due to *tsc2* deletion impairs the surface expression of Agp3 under the nitrogen depletion condition.

Altogether, these results suggest that nitrogen depletion decreases Tor2 activity through Tsc2, thereby promoting the surface expression of Agp3 from trans-Golgi/endosomes.

## Discussion

The availability of extracellular amino acids is well known to alter the functions of amino acid transporters; however, the underlying mechanism remains elusive. We and others have previously shown that Tor2 activity suppresses the transcription of amino acid transporters [13], and that this function of Tor2 is suppressed under nitrogen depletion. Here, we have provided evidence demonstrating that Tor2 activity regulates the localizations of several, but not all, intracellular amino acid transporters in both nitrogen-rich and nitrogen-depleted conditions. In the nitrogen-rich condition, Tor2 activity was found to be critical for the retention of Agp3 as well as Isp5, Aat1, and Put4 at trans-Golgi/endosomes. Furthermore, Tor2 inhibition, via treatment with a TOR inhibitor or use of the *tor2*-*287* mutant, induced the transfer of these transporters to the vacuoles, perhaps to the vacuolar lumen. On the other hand, under nitrogen depletion, reduced Tor2 activity promoted the transfer of Agp3 from the trans-Golgi/endosomes to the plasma membrane. Similarly, artificial Tor2 activation via the use of constitutively active Tor2 mutants or the *tsc2* deletion mutant maintained Agp3 localization at the trans-Golgi/endosomes. Although further study is required to determine whether YFP-fused proteins fully reflect the localizations of endogenous amino acid transporters, our findings nevertheless suggest that Tor2 activity can promote the retention of Agp3 at trans-Golgi/endosomes regardless of the availability of nitrogen sources.

The mechanism by which Tor2 regulates the localizations of Agp3 and other intracellular transporters remains elusive. The vacuolar localization of Tor2 has been documented in fission yeast [27]; however, an image of Tor2 localization deposited in the Pombase database shows rather diffuse localization throughout the cytoplasm (http://www.riken.jp/SPD/Img_page/34_iP/34C09_Loc.html). Therefore, Tor2 localization appears to vary depending on experimental conditions. In mammalian cells, mTOR has been shown to be present not only at the lysosomes but also at the Golgi apparatus [28–30]. Specifically, treatment of amino acids to HEK293E cells after amino acid starvation induced mTORC1 binding with its activator Rheb specifically at the Golgi apparatus. Therefore, Tor2 could be present at the Golgi apparatus in fission yeast, thereby functioning to retain amino acid transporters at this organelle. To elucidate the TORC1-mediated regulation of amino acid transporters, it is necessary to identify an effector molecule that is phosphorylated by Tor2 to regulate the localizations of amino acid transporters.

Tor2 inhibition was found to release Agp3 from the trans-Golgi/endosomes, whereas Agp3 was transferred to different compartments in nitrogen-rich and nitrogen-depleted conditions: the vacuolar lumen and the plasma membrane, respectively. This finding suggests that another unidentified factor regulated by nitrogen availability determines the destination of Agp3 transfer upon Tor2 inhibition. Tsc2 negatively regulates not only Tor2 but also the physical interaction between the ubiquitin ligase Pub1 and the β-arrestin-like protein Any1 [3]. Interestingly, deletion of the gene encoding either Pub1 or Any1 induces surface expression of the amino acid transporter Aat1 in fission yeast [3]. Since Tsc2 is involved in the surface expression of Agp3 upon nitrogen depletion, it may also dissociate the Pub1–Any1 interaction under this condition. This Pub1–Any1 dissociation could direct the transfer of Agp3 to the plasma membrane once it is released from trans-Golgi/endosomes by concomitant Tor2 inhibition under nitrogen depletion. By contrast, in the nitrogen-rich condition, intact Pub1–Any1 activity could suppress the surface expression of Agp3, so that Agp3 released from trans-Golgi/endosomes upon Tor2 inhibition would be transferred only to the vacuolar lumen. These possibilities warrant more detailed investigation.

In conclusion, Tor2 plays a pivotal role in regulating the localization of several intracellular amino acid transporters under both nitrogen-rich and nitrogen-depleted conditions. Since Tor2 also regulates the transcription of most of these transporters, this protein orchestrates multiple mechanisms to regulate amino acid uptake, depending on the extracellular availability of amino acids. Although Tor2 inhibition did not affect the localizations of Can1, Fnx1, or Fnx2 in this study, its role in regulating other properties of these transporters also remains to be verified.

## Supporting Information

S1 Fig Can1 and Fnx2 are localized on the vacuolar membrane. The wild-type (WT) cells expressing YFP-fused Can1 (KP6655) or Fnx2 (KP6159) protein were grown, treated, and observed as described in the legend to Fig 1, except that the cells were shifted from EMM to YES medium for 2 h before vehicle (Veh) or Torin–1 (Torin) treatment. Scale bar, 10 μm.
(TIF)

S2 Fig Torin–1 treatment induces the transfer of Agp3 from the cytoplasmic dot-like structures to the vacuolar lumen. The wild-type (WT) cells expressing YFP-fused Agp3 (KP6154) were grown, treated, and observed as described in the legend to Fig 1, except that the cells were shifted from EMM to YES medium for 2 h before vehicle (Veh) or Torin–1 (Torin) treatment. Scale bar, 10 μm.
(TIF)
