# Supplementary figures and images for: Constitutive Tor2 Activity Promotes Retention of the Amino Acid Transporter Agp3 at Trans-Golgi/Endosomes in Fission Yeast
Source: PLoS One. 2015 Oct 8;10(10):e0139045. doi: 10.1371/journal.pone.0139045 (PMC4598100; doi:10.1371/journal.pone.0139045)

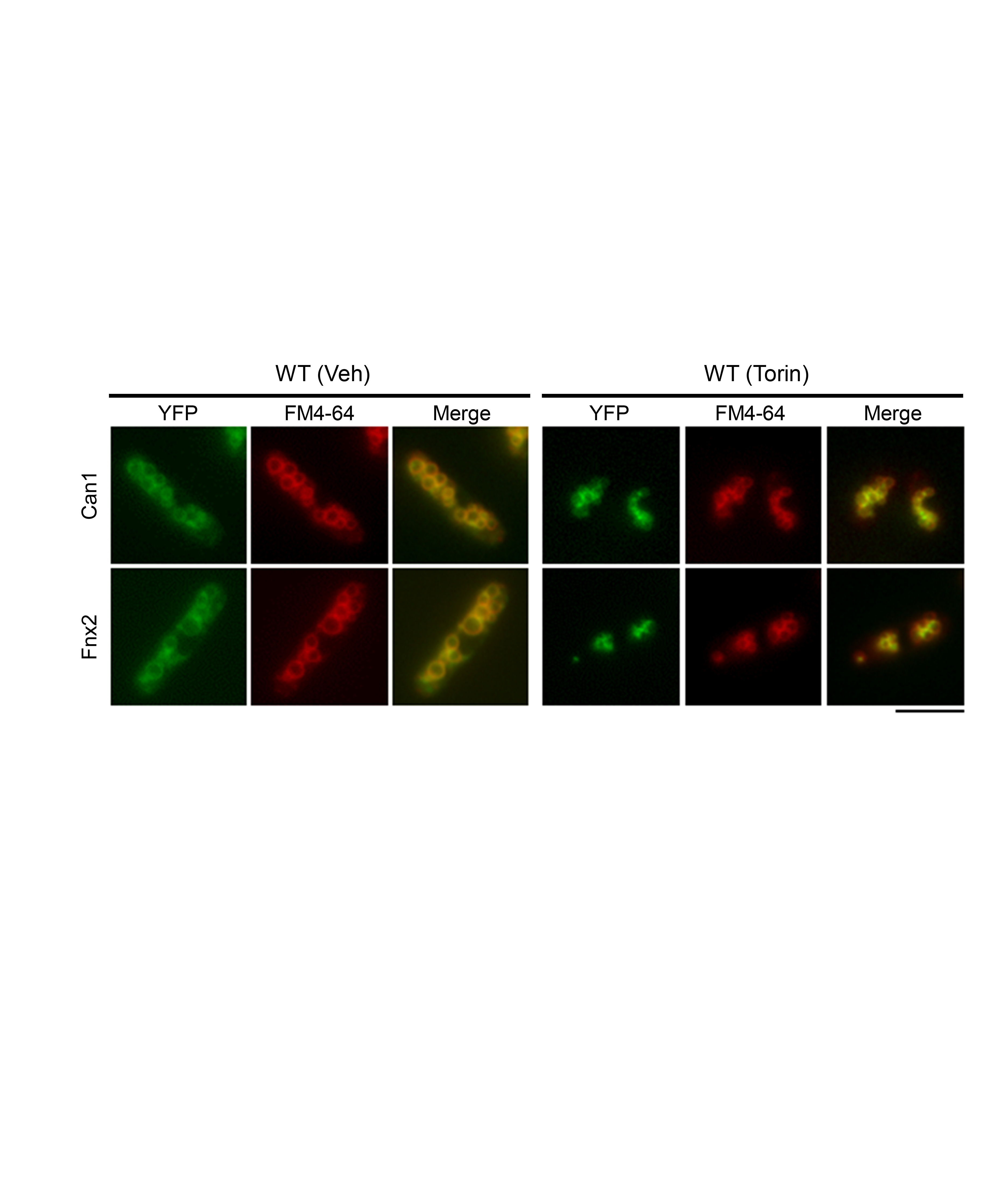

Supplement: S1 Fig — The wild-type (WT) cells expressing YFP-fused Can1 (KP6655) or Fnx2 (KP6159) protein were grown, treated, and observed as described in the legend to Fig 1, except that the cells were shifted from EMM to YES medium for 2 h before vehicle (Veh) or Torin–1 (Torin) treatment. Scale bar, 10 μm. (TIF) [file pone.0139045.s001.tif]

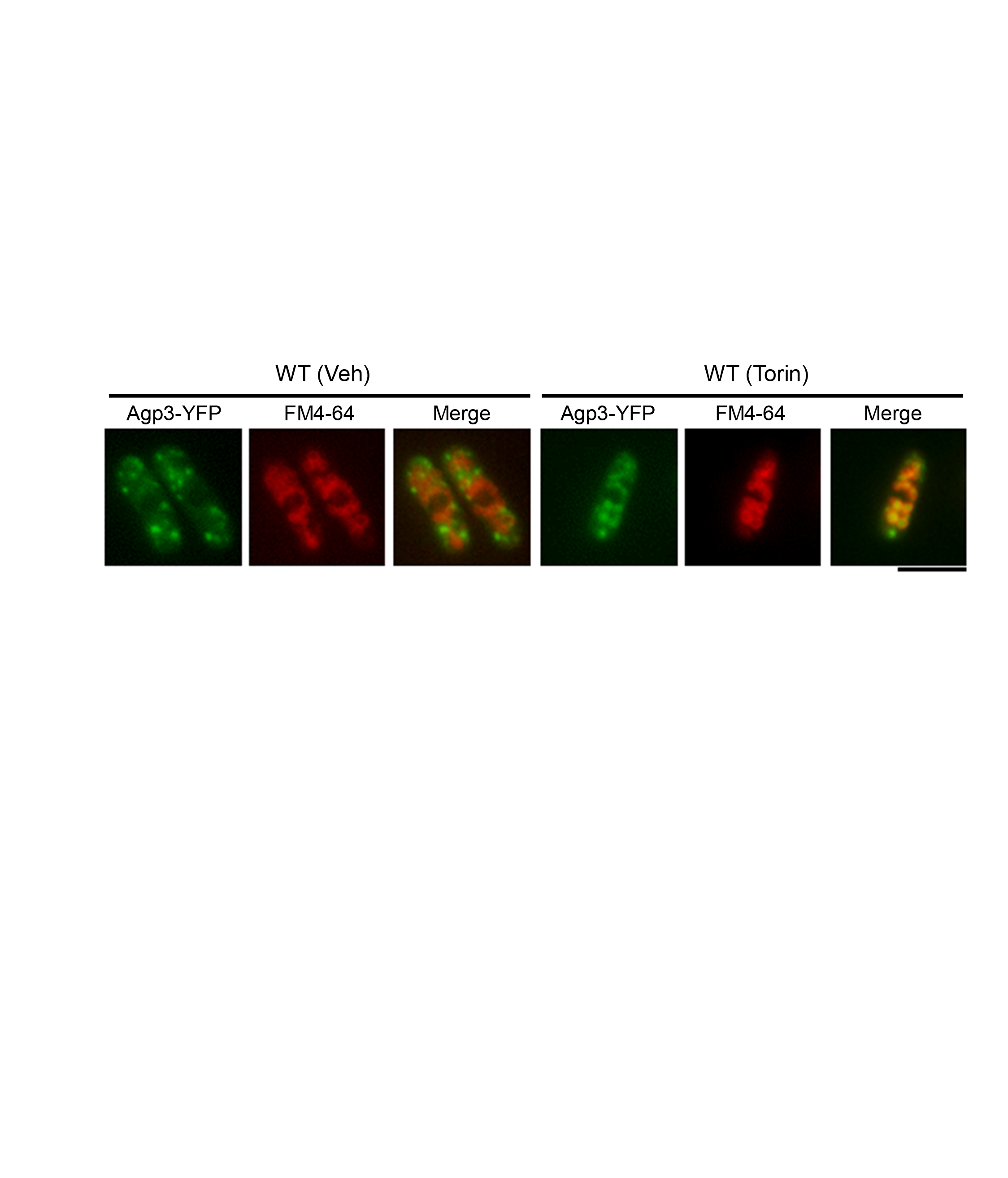

Supplement: S2 Fig — The wild-type (WT) cells expressing YFP-fused Agp3 (KP6154) were grown, treated, and observed as described in the legend to Fig 1, except that the cells were shifted from EMM to YES medium for 2 h before vehicle (Veh) or Torin–1 (Torin) treatment. Scale bar, 10 μm. (TIF) [file pone.0139045.s002.tif]
